# An Unusual Case of Esophageal Hyperplastic Polyp with Scleroderma: A Case Report and Review of the Literature

**DOI:** 10.7759/cureus.12500

**Published:** 2021-01-05

**Authors:** Zahid Ijaz Tarar, Veysel Tahan, Feng Yin, Ebubekir Daglilar

**Affiliations:** 1 Internal Medicine, University of Missouri-Columbia, Columbia, USA; 2 Gastroenterology & Hepatology, University of Missouri-Columbia, Columbia, USA; 3 Pathology, University of Missouri-Columbia, Columbia, USA

**Keywords:** dysphagia, esophagus, polyp, heartburn, scleroderma

## Abstract

Hyperplastic polyp of the esophagus is a rare condition and most of the time asymptomatic. We report a case of a 69-year-old female with scleroderma who presented with worsening dysphagia, regurgitation of food, and non-cardiac chest pain. Upper endoscopy showed a nodular lesion in the distal esophagus. Biopsy of the lesion showed features of hyperplastic polyp without any metaplastic changes. She was started on anti-acid therapy and an outpatient upper endoscopy was performed which showed no residual polyp.

## Introduction

Benign esophageal tumors are uncommon as compared to gastroesophageal reflux disease and malignant lesions. The majority of benign tumors are asymptomatic, slow growing, discovered incidentally, and have a low malignant potential, most require nothing more than diagnosis and occasional surveillance. However large benign tumors that become symptomatic require removal [1-3]. Benign esophageal tumors account for less than 1% of all esophageal tumor and 10% of all surgically resected tumors [1,2]. Benign esophageal lesions can be classified based on location (intraluminal, intramural or extramural), histological cell types (epithelial, sub epithelial or heterotopic) or based on endoscopic appearance (cystic, flat or raised) [1,4,5]. We report a patient with scleroderma who presented with dysphagia and was found to have esophageal hyperplastic polyp.

## Case presentation

**Figure 1:**
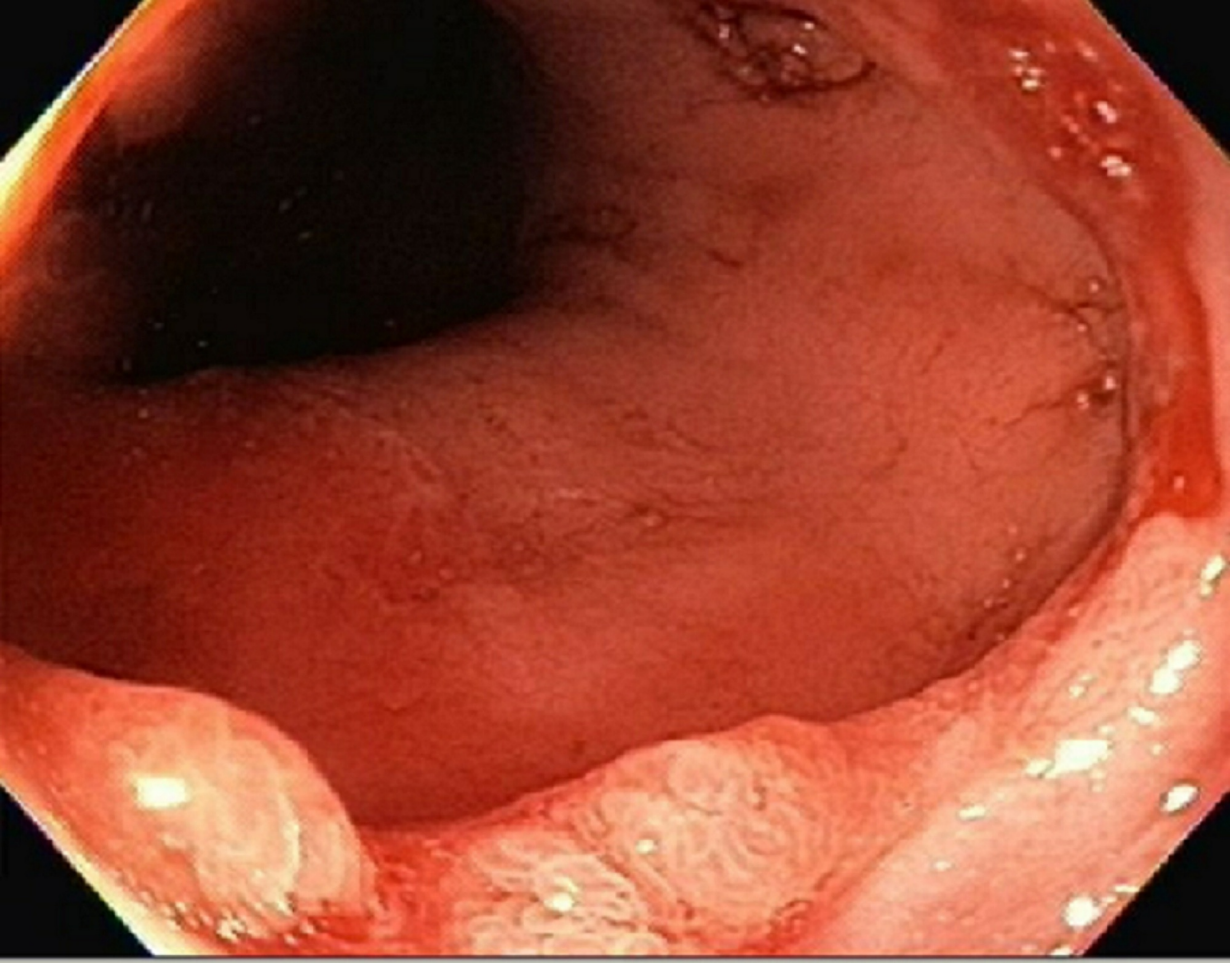
Hyperplastic esophageal polyp on a Schatzki’s ring

**Figure 2:**
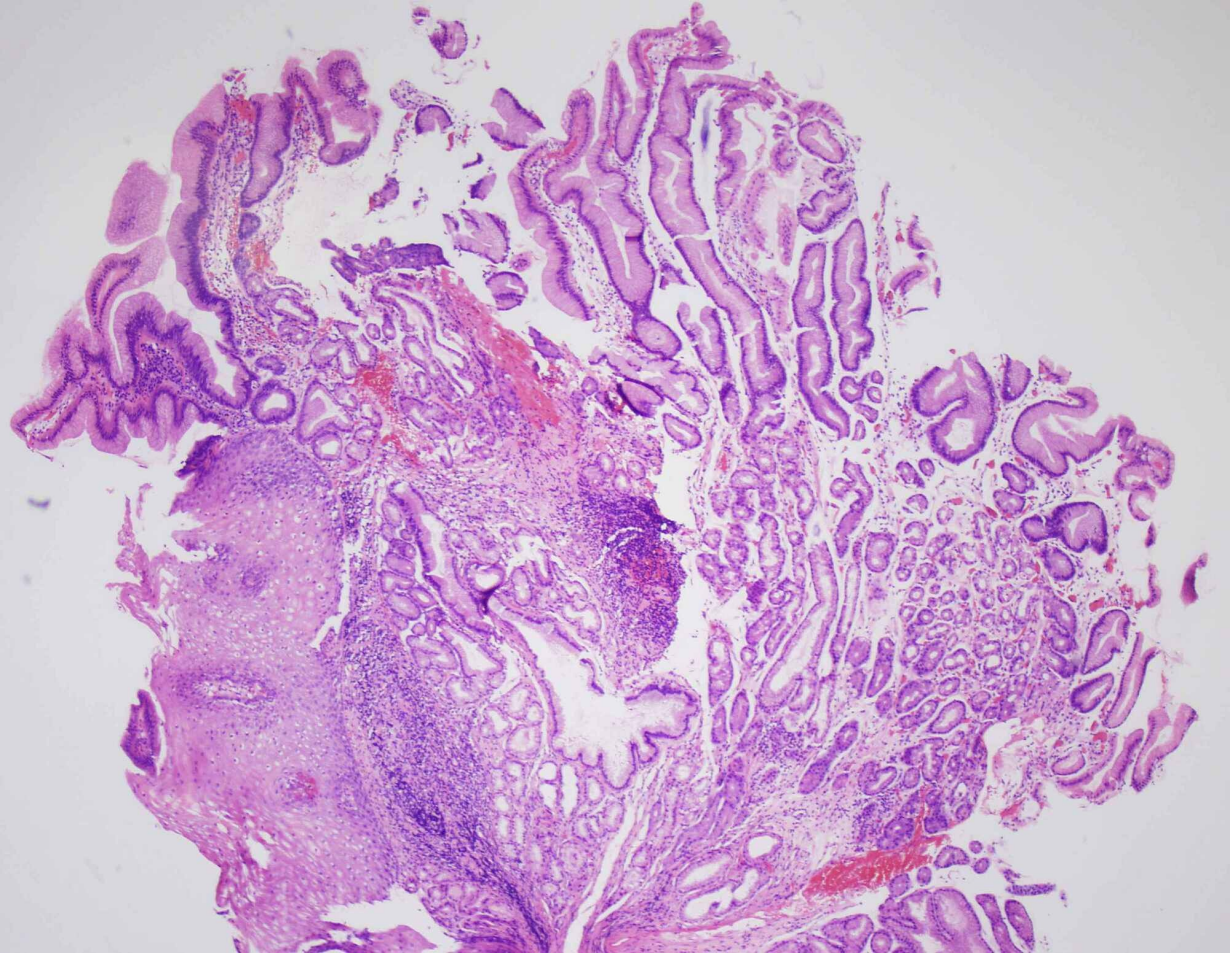
Hyperplastic polyp on Schatzki’s ring (hematoxylin and eosin stain 40x)

**Figure 3:**
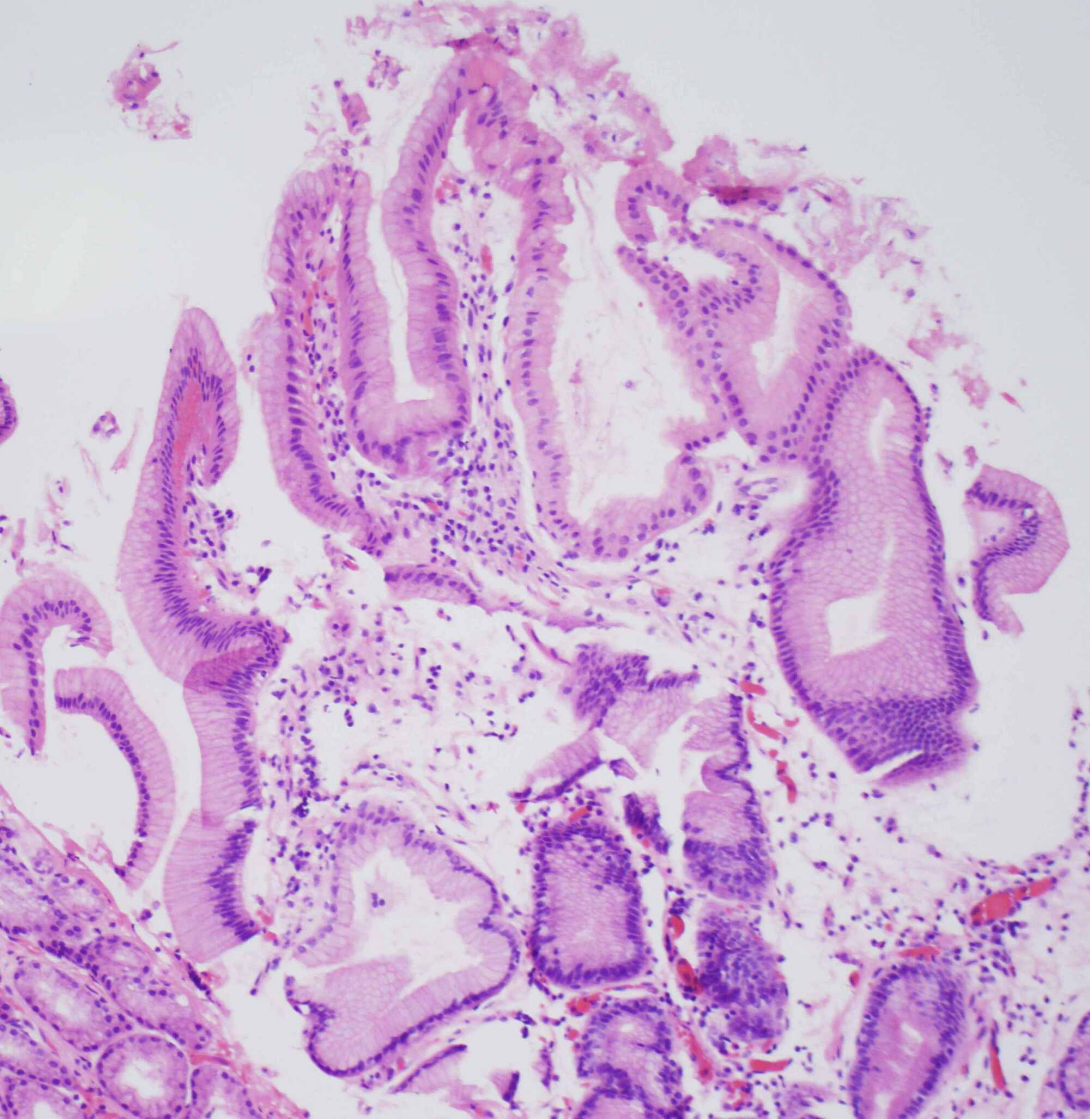
Esophageal hyperplastic polyp on Schatzki’s ring (hematoxylin and eosin stain 100x)

A 69-year-old female with a medical history of scleroderma was referred by her primary care physician for evaluation of worsening solid food dysphagia. She had dysphagia for many years which got worse lately and was associated with regurgitation of food, and non-cardiac chest pain. She had no problem drinking fluids. There was no associated weight loss, night sweats, nausea, vomiting or diarrhea. Physical examination was notable for diffuse skin tightening consistent with known diagnosis of scleroderma but was otherwise unremarkable. An esophagogastroduodenoscopy (EGD) was performed to investigate her symptoms which revealed a 3-cm sliding type hiatal hernia and a Schatzki’s ring. Following a careful examination of the distal esophagus and the ring, a subtle nodularity was detected (Figure 1). The ring was broken with a Savary 48 Fr dilator. Biopsies were obtained from the nodularity separately with biopsy forceps for histopathological examination which showed squamo-columnar mucosa with elongated and irregular hyperplastic foveolar epithelium with cystic dilatations, and acute and chronic inflammatory infiltrates with edematous changes were detected in lamina propria, which were morphologically consistent with esophageal hyperplastic polyp. There was no evidence of intestinal metaplasia or dysplasia (Figures 2-3). She was started on high dose proton pump inhibitor therapy and follow-up EGD was performed after two months which did not show the polyp.

## Discussion

Hyperplastic polyps of the esophagus and esophagogastric junction (EGJ) are uncommon lesions characterized by hyperplastic epithelium (foveolar, squamous or mixed), with variable amount of stroma [5,6]. Hyperplastic polyps are most commonly located in the region of EGJ, followed by the distal esophagus and mid esophagus [4,5]. The pathogenesis of hyperplastic polyp is not clear but majority of reported cases have occurred in association with gastroesophageal reflux disease (GERD) [5,7]. There are other reports which showed hyperplastic polyp association with vomiting [5,8], Crohn’s disease [5,9], sclerotherapy [5,10] heterotopic gastric mucosa [5,11,12], and Barrett’s esophagus [5,13,14]. Hyperplastic polyp was associated with GERD in 48% of the cases [5]. Other likely causes are medication induced esophagitis, infection, anastomosis or polypectomy sites, and photodynamic therapy [5]. In the pediatric age group, there are only few reported cases of esophageal polyps out of which three showed association with NF type 1 [6,15]. Two studies have shown the association of esophageal hyperplastic polyp with Barrett’s esophagus, although both have conflicting evidence. Abraham et al. reported a series of 30 hyperplastic polyps in 27 patients, and out of these, four (15%) patients had underlying Barrett’s esophagus [5]. On the other hand, Long and Odze reviewed 134 GEJ polyps, out of which 46 were hyperplastic and they compared them with 46 hyperplastic polyps of the gastric antrum and corpus, 15 (33%) of these esophageal hyperplastic polyps were associated with Barret esophagus [13]. Tsai et al. studied endoscopic features of 2997 patients, 149 patients had benign esophageal lesions, which were further divided into epithelial (129) and sub-epithelial lesions (20) based on histological findings. Out of epithelial lesions, 18 were histologically proven hyperplastic polyps with a higher incidence in men (12) as compared to women (6). Other epithelial lesions were glycogenic acanthosis, heterotopic gastric mucosa, squamous papilloma, ectopic sebaceous gland, and xanthoma [4]. Hyperplastic polyps are more common in the stomach as compared to the esophagus. Buyukasik et al. reviewed 55,897 endoscopies from 2006 to 2012 and found 66 cases of upper gastrointestinal polyps in 59 patients; 44 (67%) of these polyps were hyperplastic based on histology. Most common location of these polyp was in gastric antrum 29 (43%), followed by corpus 15 (23%), cardia 11 (16.7%), fundus 3 (4.6%), bulbous 2 (3%), second part of duodenum 3 (4.6%) and only three were in the lower esophagus (4.6%) [16]. There are no reported cases of malignant transformation of esophageal hyperplastic polyp but there are studies and reported cases of malignant transformation of gastric and colonic hyperplastic polyps [17,18]. Heterotopic gastric mucosa has the potential for transformation into adenoma and adenocarcinoma, but there are no reported cases of malignant transformation of polyps arising in heterotopic gastric mucosa in esophagus [12]. Heterotopic gastric mucosa is seen in the cervical esophagus and is congenital most of the time due to incomplete replacement of original columnar epithelium by stratified squamous epithelium [12,19] or it can be acquired in response to a chronic local stimulus (i.e cigarette smoke, alcohol, regurgitation of stomach acid [20]. Benign esophageal lesions have a wide spectrum of clinical and pathological features. Understanding of endoscopic and pathological features of esophageal lesions is essential for their detection, diagnosis, and management [4]. Patients with hyperplastic polyp of the esophagus need to be observed regularly to be excised if causing symptoms or growing in size [20]. Hyperplastic polyp can regress after anti-acid therapy [4]. Endoscopic mucosal resection is recommended for symptomatic polyps and those associated with Barrett esophagus [4,14].

In our case, following removal of the polyp with forceps biopsy, we initiated anti-acid therapy. Follow-up endoscopy in two months was negative for polyp recurrence and the patient's symptoms significantly improved. This case has previously been presented as an abstract (Abstract: Tarar Z, Tahan V, Daglilar E, Basar O, Yin F. A Rare Case of Hyperplastic Polyp of Esophagus on a Schatzki's Ring. Annual Scientific Meeting, American College of Gastroenterology; October 23-28, 2020, Nashville, Tennessee).

## Conclusions

Hyperplastic esophageal polyps are rare and most commonly associated with GERD. To our knowledge, we report the second case of esophageal hyperplastic polyp that was grown on Schatzki's ring. These polyps need to be observed closely. They regress with anti-acid therapy in most cases. Endoscopic mucosal resection is recommended for symptomatic polyps and those associated with Barrett esophagus.
